# Is It Possible to Achieve Favorable Accelerated Dental Changes with No Periodontal Complications When Retracting Upper Anterior Teeth Assisted by Flapless Corticotomy Compared to Traditional Corticotomy? A Two-Arm Randomized Controlled Trial

**DOI:** 10.1155/2022/4261248

**Published:** 2022-03-07

**Authors:** Hanin Nizar Khlef, Mohammad Younis Hajeer

**Affiliations:** Department of Orthodontics, University of Damascus Dental School, Damascus, Syria

## Abstract

**Objectives:**

The objective of this trial was to evaluate the dental changes, periodontal health, and tooth vitality in mini-screw-supported *en-masse* retraction with two corticotomy-based acceleration techniques. *Study Design*. The sample included 38 adult patients presenting with class II division 1 malocclusion (three males, 35 females; age range between 18 and 30 years), needing the extraction of upper first premolars followed by *en-masse* retraction. The sample was divided randomly and equally into two groups. Randomization was carried out by random numbers generated by the computer with a 1 : 1 allocation ratio. The allocation concealment was carried out by sequentially numbered, opaque, sealed envelopes. The interventions were traditional corticotomy (TC) versus flapless corticotomy (FC). Mini-screws were inserted between the upper second premolar and first molar, bilaterally. The primary outcome was evaluating dental changes. Secondary outcomes were the periodontal health and pulp vitality of the maxillary teeth. Mann–Whitney *U* test and two-sample *t*-test with Bonferroni correction were used to analyze the data.

**Results:**

The *en-masse* retraction rate in the first three months was higher in the TC group than the FC group (1.82, 1.66, and 1.39 mm/month vs 1.60, 1.42, and 1.22 mm/month, respectively) with statistically significant differences (*P* < 0.001, *P* < 0.001, *P*=0.001, respectively). The *en-masse* retraction amount was greater in the TC group than the FC group (6.84 mm vs 6.18 mm, respectively) with statistically significant differences (*P*=0.002). There was an increase in the inter-canine and inter-molar widths with a minor distal movement of the upper first molar in the two groups, with no significant differences between them (*P* > 0.008). The values of gingival, papillary bleeding and plaque indices in the TC group were significantly greater than those in the FC group after performing the corticotomy (*P* < 0.001, *P* < 0.003, *P*=0.002, respectively). No gingival recession was found on any of the examined teeth in both groups. All teeth maintained their vitality at all measurement times in both groups. No severe harms were noticed in any group.

**Conclusions:**

Both traditional and flapless corticotomy techniques resulted in clinically similar rates of the *en-masse* retraction of upper anterior teeth, with similar dental changes and no significant periodontal complications or tooth vitality loss. The minimally invasive flapless corticotomy appeared to be a good alternative to the more invasive traditional corticotomy. This trial is registered with https://www.clinicaltrials.gov (Identification code: NCT04847492), retrospectively registered.

## 1. Introduction

The upper dental protrusion is one of the most widespread conditions in practice [1]. Management of class division 1 malocclusion is an issue of importance, as this malocclusion has a negative impact on the aesthetic and functional aspects [2]. Camouflage therapy of class division 1 relies on the premolar extraction followed by the retraction of the upper anterior teeth [3]. Numerous articles have demonstrated that the *en-masse* retraction method is more beneficial than the two-step retraction method due to several advantages, chiefly reducing the treatment duration [4]. The most widely used technique for *en-masse* retraction is the sliding technique, which is often supported by mini-screws to obtain absolute anchorage [5].

One of the considerable challenges in the profession is prolonged orthodontic treatment duration [6]. For that, many methods have been suggested to minimize orthodontic treatment time as the surgical interventions [7]. Even though the traditional corticotomy with flap elevation substantiated to be efficient in accelerating several species of tooth movement [8], it has been considered invasive [9]. So, minimally invasive surgical procedures have been suggested and labelled “flapless corticotomies” [10]. A recent randomized clinical trial by Khlef et al. [11] found that the average *en-masse* retraction time for the traditional corticotomy group was 3.75 months, versus 4.04 months for the flapless corticotomy group, with no significant difference between both corticotomy techniques. As a result, they concluded that both traditional and flapless corticotomies carried out by piezosurgery effectively accelerated the *en-masse* retraction of the upper anterior teeth. On the other hand, their trial did not provide information about the *en-masse* retraction rate per month, the anteroposterior and horizontal movement of canines and first molars during the retraction, and the effect of corticotomy procedure on the periodontal tissues as well as tooth vitality.

One of the frequent complications during fixed orthodontic treatment is the periodontal problems since the fixed orthodontic device allows the accumulation of bacterial plaque around its elements [12]. Bacterial plaque is the primary factor in the initiation, progression, and even re-emergence of periodontal problems [13]. Aboul-Ela et al. [14] study showed a rise in the grade of gingivitis following the traditional corticotomy due to the gingival reaction in conjunction with the bone healing process. On the other hand, Abed and Al-bustani [15] study found no bleeding on probing after the traditional corticotomy, which indicated the absence of gingivitis. Regarding the flapless corticotomy, Cassetta et al. [16] found an improvement in the gingival index following the surgical procedure. In contrast, Charavet et al. [17] and Aksakalli et al. [18] studies found a regression in the assessed periodontal indices after the corticotomy, with no significant differences between before and after the surgical procedure. Three case reports [19–21] and two canine retraction studies [15, 22] evaluated the vitality of the teeth adjacent to the corticotomy, and they found no loss of tooth vitality, taking into account that all these articles except one [15] did not mention the employed method for tooth vitality evaluation.

Reviewing the literature reveals that there is no randomized controlled trial (RCT) evaluating the *en-masse* retraction of upper anterior teeth in conjunction with traditional or flapless corticotomy regarding dentoalveolar changes, periodontal health, and pulp vitality of maxillary teeth.

So, the purposes of this study were to compare the traditional and flapless corticotomies in terms of (1) speed of retraction, (2) the dentoalveolar changes of the maxillary dental arch, (3) the periodontal health, and (4) the pulp vitality of maxillary teeth. The null hypotheses stated that there were no differences between the two corticotomy techniques in (1) the speed of *en-masse* retraction of upper anterior teeth, (2) the dentoalveolar changes of the maxillary dental arch, (3) the periodontal health, and (4) the pulp vitality of maxillary teeth.

## 2. Materials and Methods

### 2.1. Trial Design, Registration, and Any Changes following Trial Commencement

The report of this study was written according to CONSORT (Consolidated Standards of Reporting Trials) statements. It was a single-centre, two-arm parallel group randomized controlled trial. This study was listed in the database of https://clinicaltrials.gov (Identification code: https://clinicaltrials.gov/ct2/show/NCT04847492), and it was funded by the Damascus University Postgraduate Research Budget (Grant number 92365814682DEN). This trial was performed conforming to the guidelines of the Helsinki Declaration and was approved by the Local Ethics Committee at Damascus University Dental School, Syria (Ref no: UDDS-527-09062017/SRC-3502). No changes were made in the study protocol following trial commencement.

### 2.2. Participants, Eligibility Criteria, and Settings

**Figure 1:**
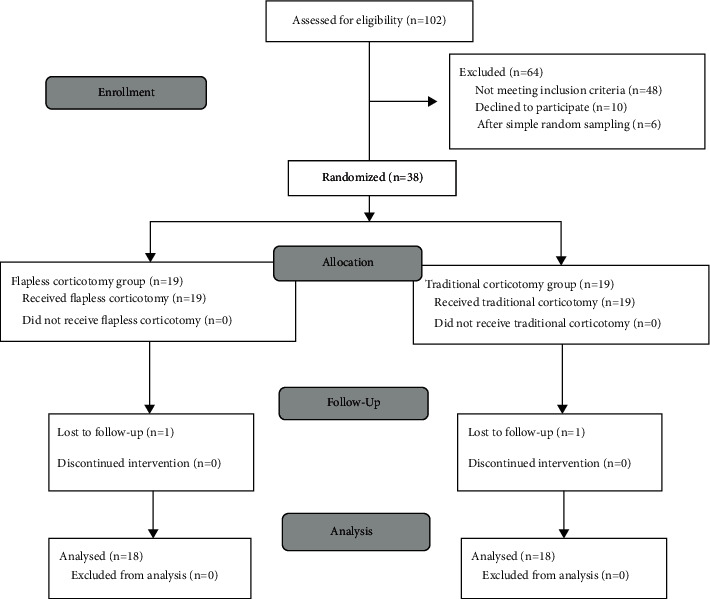
CONSORT (Consolidated Standards of Reporting Trials) participants' flow diagram.

Thirty-eight adult patients (three male, 35 female) were selected from the Department of Orthodontics, xxxxxxxx University Dental School, xxxxx, from June 2017 to January 2018. A flowchart of patients' allocation and follow-up is shown in Figure 1.

The participants were distributed randomly and equally into two groups: flapless corticotomy group (FCG = 19) and traditional corticotomy group (TCG = 19). The criteria for including patients were as follows: (1) class II division 1 malocclusion needing the extraction of upper first premolars, (2) age range between 18 and 30 years, (3) skeletally, mild to moderate class II malocclusion, (4) excessive or average anterior facial height, (5) no or slight crowding (tooth-size arch-length discrepancy ≤3 mm), (6) full-permanent dentition (with the exclusion of third molars), (7) overjet >5 mm and <10 mm, (8) shallow or normal overbite (30% or less), (9) no history of any orthodontic treatment, (10) no any disease or medicine intaking that may impact the bone and/or tooth movement, and (11) healthy periodontal tissues and good level of oral hygiene.

The candidate participants were particularly informed about the trial interventions and were asked if they intended to take part in the trial. An information sheet was distributed to all participants, and they were asked to sign an informed consent prior to trial recruitment. No changes in the techniques were carried out after starting the trial commencement.

### 2.3. Interventions

#### 2.3.1. Levelling and Alignment Phase

The first premolars extraction was performed at the onset of the orthodontic treatment for all patients. 0.022 × 0.028-inch brackets (Votion, Ortho Technology, Tampa, Fla) were used to treat the patients. Self-drilling titanium mini-screws (1.6 mm in diameter, 8 mm in length; 3S screw, Hubit™, Seoul, Korea) were inserted 8–10 mm above the archwires between the upper second premolar and first molar bilaterally, and these teeth were attached to the mini-screw with a ligature wire. Patients were given instructions for oral hygiene by brushing their teeth according to the modified Bass technique [23]. Patients were recommended to use a manual medium toothbrush with toothpaste containing fluoride three times a day. Also, they were instructed to use interdental brushes and mouthwashes twice a day. The sequence of archwire replacement was as follows: 0.014-inch nickel-titanium (NiTi), 0.016-inch NiTi, 0.016 × 0.022-inch NiTi, 0.017 × 0.025-inch NiTi, 0.019 × 0.025-inch NiTi, and 0.019 × 0.025-inch Stainless Steel (SS) [4]. The archwires were changed every three weeks during this phase.

#### 2.3.2. The Flapless Corticotomy Procedure

**Figure 2:**
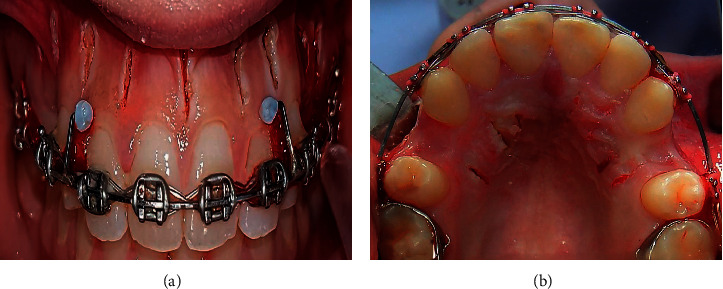
The flapless corticotomy from the buccal view (a) and the palatal view (b).

In the FGC, vertical soft tissue cuts were performed on the palatal and buccal gingiva. One cut was performed between the six maxillary anterior teeth roots, and two cuts were performed between the upper canines and second premolars. The cuts were 5 mm long and started 4 mm above the interdental papilla. Then, a piezoelectric knife was used to make incisions in the cortical alveolar with 3 mm in depth and 8 mm in length. No surgical suturing was needed (Figures 2(a) and 2(b)).

#### 2.3.3. The Traditional Corticotomy Procedure

**Figure 3:**
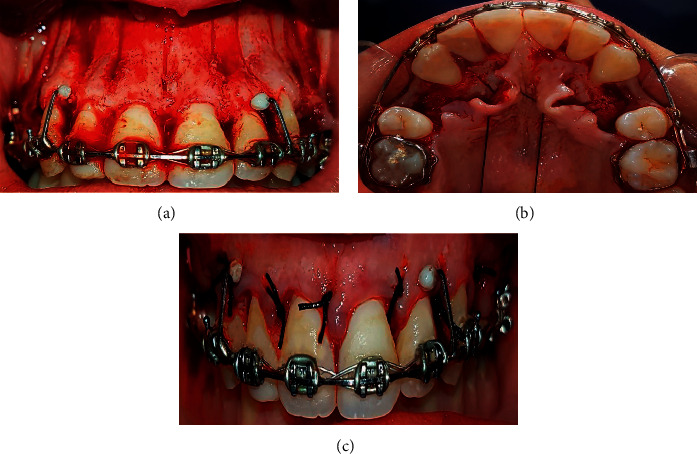
The traditional corticotomy from the buccal view (a) from the palatal view (b) and the surgical flap suturing (c).

In the TCG group, a full-thickness mucoperiosteal flap was lifted from the palatal and buccal sides. Then, the piezoelectric knife was used to perform one vertical incision between the upper anterior teeth roots and two vertical incisions at the extraction site. A horizontal incision connected the vertical ones. The vertical incisions were 3 mm in depth, started 2 to 3 mm apical to the alveolar crest, and extended 3 mm over the root apices (Figures 3(a) and 3(b)). Finally, the interrupted technique of suturing was carried out (Figure 3(c)).

#### 2.3.4. Postcorticotomy Phase

Following the surgery, all patients were asked to (1) take antibiotic tablets (Augmentin 1000 mg), one tablet twice a day for one week, (2) put ice packs on, for 6 to 8 hours following surgery, (3) keep a high level of oral hygiene, (4) eat soft food for some days following surgery, (5) stop smoking for the first week after surgery, and (6) take acetaminophen 500 mg when required to relieve pain.

#### 2.3.5. The *En-Masse* Retraction Phase

The retraction was initiated four days following the corticotomy procedure using 0.019 × 0.025-inch SS archwires with 8 to 10 mm soldered hooks located distal to the lateral incisors. NiTi closed coil springs (American Orthodontics, Wisconsin, USA) with 9 mm length were extended from the soldered hooks to the mini-screws, and 250 g of force for each side was applied. The patients' follow-up sessions were every two weeks during the *en-masse* retraction phase [24]. The force was measured on every appointment and adjusted if needed. The endpoint of the monitoring time was when obtaining a class I canine's relationship and a correct incisor's relationship.

### 2.4. Primary and Secondary Outcomes and Any Changes following Trial Commencement

The primary outcomes were the *en-masse* retraction rate of upper anterior teeth, the molar's anteroposterior movement, and inter-molar and inter-canine widths in flapless and traditional corticotomy techniques. In contrast, the secondary outcomes were the gingival index, dental plaque index, papillary bleeding index, gingival recession index, and maxillary dental arch pulp vitality.

The archwires were removed before taking the alginate impression, followed by applying a ligature elastic around the brackets to ensure having accurate and nondistorting impressions. The impressions were taken at the end of the levelling and alignment stage and prior to starting the *en-masse* retraction (T0), one month (T1), two months (T2), three months (T3), four months (T4), and five months (T5) after the start of *en-masse* retraction. The final impression was taken at the termination of *en-masse* retraction (when obtaining a class I canine's relationship and a correct incisor's relationship) [4].

**Figure 4:**
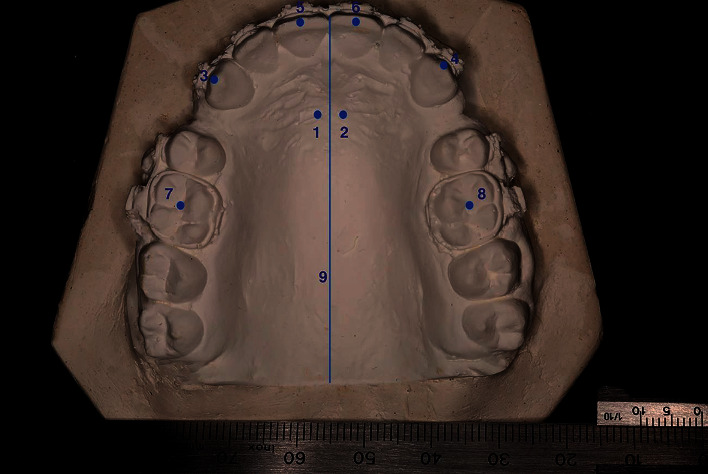
Landmarks used on study models. (1) The medial end of the right third palatal ruga, (2) the medial end of the left third palatal ruga, (3) the cusp of the right maxillary canine, (4) the cusp of the left maxillary canine, (5) the middle of the incisal edge of the right maxillary central incisor, (6) the middle of the incisal edge of the left maxillary central incisor, (7) the central fossa of the maxillary right first molar, (8) the central fossa of the maxillary left first molar, (9) the midpalatal suture line.

**Figure 5:**
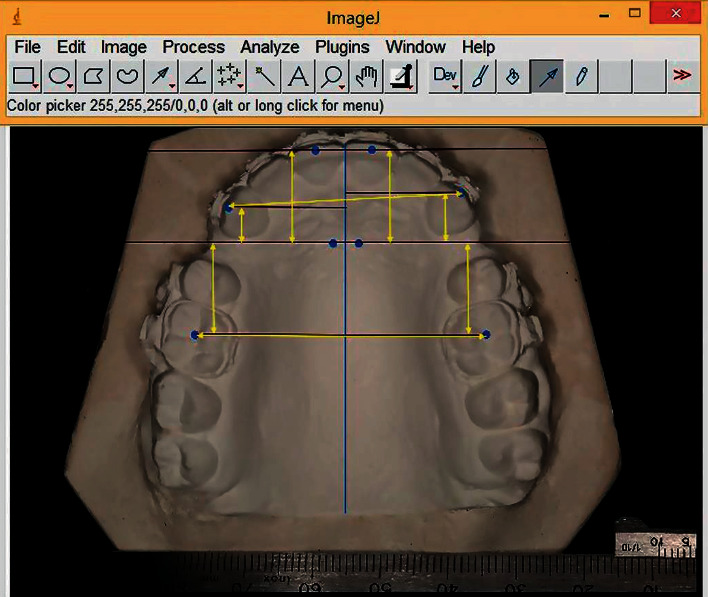
Measurements on digital photographs using the ImageJ program.

Maxillary casts were photographed digitally using a Canon EOS 5D Mark III, Lens type, with the dimensions Macro Medium Telephoto Canon EF, 100 mm, f/2.8 L Macro IS USM (Canon, Tokyo, Japan). The photography was accomplished by focal projection perpendicular to the occlusal plane. A metal millimetre ruler was put next to the study cast for calibration. The measurements were taken from the digital photographs using the ImageJ program as the way stated by Al-Imam et al. [25]. The landmarks are shown in Figure 4, and the measured variables are shown in Figure 5.

The periodontal health was evaluated by measuring the following parameters: dental plaque and gingival indices according to Silness and Loe [26, 27], papillary bleeding index according to Muhlemann [28], and gingival recession index according to Miller [29]. The cold test was applied to examine the vitality of the upper teeth from the right first molar to the left first molar using ethyl chloride spray (endo ice) at a temperature of −50°. A small amount of ethyl chloride was applied to a small cotton ball at the cervical third of the crown. The cotton ball was kept in contact with the tooth until the patient's pain response occurred, considering that the endo ice application should not exceed 5 seconds. The periodontal indices and tooth vitality were assessed within the following times: before the commencement of orthodontic treatment (T0), after the fulfilment of the levelling and alignment stage and before the surgical intervention (T1), and following the completion of the *en-masse* retraction (T2). No outcome changes following trial commencement.

### 2.5. Sample Size Calculation

The sample size calculation was made by using Minitab® (version 17, State College, Pennsylvania, USA), presuming that a 0.25 mm is the least significant difference to be detected between the two corticotomy methods in the *en-masse* retraction rate of maxillary anterior teeth, with taking into account that the standard deviation of this variable was 0.2225 (based on Sakthi et al. [30] study). When paired *t*-tests were employed with a 5% alfa level and 80% power, at least 18 participants per group were desired. To compensate for any possible participants' loss (not more than 5%), 19 participants were needed per group.

### 2.6. Randomization, Allocation Concealment, and Implementation

Simple randomization was carried out by an academic colleague using random numbers generated by the computer with a 1 : 1 allocation ratio. The allocation sequence was hidden using sequentially numbered, opaque, sealed envelopes. These envelopes were opened following the levelling and alignment stage.

### 2.7. Blinding

Blinding was impossible for both patients and investigators carrying out the clinical procedures (xxxx), while blinding was performed only for the assessor of outcomes (xxxx).

### 2.8. Statistical Analysis

Two statistical programs were used for data analysis: the SPSS® program (version 21; IBM®, Armonk, NY, USA) and Minitab® (version 17, State College, Pennsylvania, USA). Anderson–Darling normality tests were applied to check the normality of distributions. The Mann–Whitney *U* test and two-sample *t*-test with Bonferroni correction were utilized.

### 2.9. The Error of the Method and Assessment of Reliability

After a four-week interval, twenty dental casts were randomly chosen (ten out of each group). Digital photography for dental models has been retaken with the same previous conditions. Following this, all reference lines and points were re-determined, and measurements were re-done by the ImageJ program. The interclass correlation coefficients (ICCs) were applied to define any random error, whereas paired-sample *t*-tests were utilized to detect any systematic error.

## 3. Results

### 3.1. Participant Flow, Recruitment, and Baseline Data

Of the 102 subjects evaluated for the trial from June 2017 to January 2018, 48 cases did not match the inclusion criteria, ten refused to participate, and six were ruled out after simple random sampling. A total of 38 participants (three male and 35 female) were enrolled, 19 participants per group. One participant had missed the follow-up appointments in each group, leaving 18 patients per group for the data analysis stage, as mentioned in Figure 1. The main characteristics of the sample are shown in Table 1.

### 3.2. The Error of the Method

The ICCs ranged from 0.978 to 1, which pointed out a high intra-examiner reliability for the performed measurements (Supplementary Table S1). No significant difference between the two measurements was shown by paired-sample *t*-tests (*P* > 0.05). Consequently, systematic errors were minor and insignificant (Supplementary Table S2).

### 3.3. Outcomes and Estimation

#### 3.3.1. Dental Change Measurements

The *en-masse* retraction rate in the TCG in the first three months was 1.82, 1.66, and 1.39 mm/month, respectively, whereas it was 1.60, 1.42, and 1.22 mm/month, respectively, in the FGC, and the differences were statistically significant (*P* < 0.001, *P* < 0.001, *P*=0.001, respectively; Table 2). On the other hand, the *en-masse* retraction rate in the 4th and 5th months in the TCG was 1.14 and 0.89 mm/month, respectively. At the same time, it was 1.08 and 0.98 mm/month, respectively, in the FGC, without any significant differences between the two groups (*P*=0.208, *P*=0.148, respectively). The mean difference between the two groups in the amount of *en-masse* retraction was 0.66 mm, which was statistically significant (*P*=0.002). Regarding the first molars' anteroposterior movement, there was no significant difference between both groups during all evaluation times (*P* > 0.008; Table 3). There was no significant difference between the two corticotomy techniques during the three measurement times for the inter-canine and inter-molar width changes (*P* > 0.008; Table 4).

### 3.4. Periodontal Indices

There were no significant differences in the gingival, papillary bleeding, and plaque indices between the FGC and TCG at T0 and T1 (*P* > 0.017, Table 5). At the same time, there were significant differences between both groups in these indices at T2 (*P* < 0.017, Table 5). The gingival recession index showed the absence of recession at examined teeth in both groups at T0, T1, and T2.

### 3.5. Tooth Vitality

With respect to the tooth vitality, there was no significant difference between both groups, as the endo ice test showed persistent vitality of all examined teeth at all times of measurement (T0, T1, and T2).

### 3.6. Harms

No severe harms were noticed in any group.

## 4. Discussion

As far as we know, this is the first randomized controlled trial evaluating the *en-masse* retraction of upper anterior teeth assisted by traditional or flapless corticotomy interventions. The intention was to evaluate the possibility of replacing traditional corticotomy with a less invasive technique in the event of obtaining similar results regarding the speed of retraction, the dentoalveolar changes, the periodontal health, and the tooth vitality of maxillary teeth.

The upper first premolars were extracted at the onset of orthodontic treatment because the extraction might affect the tooth movement rate [31]. The mini-screw was used as a direct and indirect anchorage method [11]. NiTi closed coil springs were utilized for *en-masse* retraction instead of chain elastic due to their continuous light force [12]. A 250 g of force was used during the retraction phase, considered within the physiological range and distributed evenly on the upper anterior teeth [30]. The retraction phase was started four days after doing the corticotomy [11], with patients followed up once every two weeks to get the most benefits of the regional acceleration phenomenon (RAP) [10].

The piezosurgery device was chosen to perform both flapless and traditional corticotomy due to its advantages compared to rotary instruments, as it provides accurate and selective incision of hard tissues without hurting adjacent soft tissues, fast bone healing without necrosis due to no heat generation during the cut, ensure a clean, bloodless workspace with perfect vision, and greater patient acceptance because of less vibration and noise compared to traditional burs [32]. The medial end of the third palatine rugae was utilized as a reference mark on the study models because it is considered a stable point [10].

### 4.1. Main Findings Based on Existing Evidence and Interpretation

The current trial showed that the *en-masse* retraction rate in the first three months was higher in the traditional corticotomy group in comparison with the flapless corticotomy group since the bone and gingival harms were greater in the TCG compared to the FCG. It can be noted that the differences between the two groups were minimal in the first three months (0.23 mm in the first month (T1), 0.24 mm in the second month (T2), and 0.17 mm in the third month (T3)). Despite being statistically significant, they were clinically unimportant. Thus, it can be inferred that the traditional corticotomy is not better than flapless corticotomy in accelerating the *en-masse* retraction from the clinical perspective.

Sakthi et al. [30] found that the *en-masse* retraction rates in TCG were 1.80 mm in the first month, 2.01 mm in the second month, and 1.93 mm in the third month. These findings were greater in comparison with the current study in the second and third months, and this may be partially explained by the timing of premolars extraction, which was done in the same session of corticotomy in the study of Sakthi et al. [30] Simultaneous extraction and corticotomy may have further weakened the bone resistance to the tooth movement and increased the *en-masse* retraction rate. It was decided in the present trial to extract the maxillary first premolars at the onset of treatment to measure the pure impact of corticotomy. Additionally, the *en-masse* retraction rate in Sakthi et al. [30] study was measured by a digital calliper from the mesial maximum outline of the second premolar to the distal maximum outline of the canine. Therefore, their measurements included both the expected mesial movement of the posterior segment and the distal movement of the anterior segment, whereas, in the current study, the measurements were done according to the medial end of the third palatal ruga because it is a steady reference point for measuring the anteroposterior tooth movement [33]. Additionally, the bone injury induced in Sakthi et al. [30] study was more than that of the present study as Sakthi et al. [30] used round fissure burs to perform selective alveolar decortication. In contrast, the piezosurgery knife made linear incisions in the current study, that is, less bony injury.

The current trial results demonstrated that both groups had a minor distal movement of the upper first molars. This is due to the friction between the base archwire and the tubes of the first molar bands during the *en-masse* retraction stage [31]. Additionally, when an interdental contact occurred between the canines and second premolars, a retraction force was translated to the first molar [34]. The distal movement was simple due to the suspension of the orthodontic force once the canines arrived at a class I relationship. The results of the current trial are aligned with Khlef et al. [11] study, who noticed that the maxillary first molars were slightly distalized at the end of the retraction stage in both flapless and traditional corticotomy groups with no significant difference between them. In addition, the finding in the FGC group was consistent with that of Tuncer et al. [31], who found that the first molar showed a distal movement. However, the current study differed from that of Tuncer et al. [31] by the amount of distal movement, as the mean amount of movement was 1.17 mm in their study. Surprisingly, Sakthi et al. [30] did not apply any anchorage method in their study in both TCG and control group (no surgical intervention); however, they found a significantly lesser mesial movement of first molars compared to the control group, which might be explained that the corticotomy had weakened the bone resistance in the anterior region. Thus, it led to a greater amount of posterior anchorage.

An increase in the inter-canine and inter-molar widths was obtained after completing the *en-masse* retraction in the two groups with no significant differences between them (*P*=0.157, *P*=0.074, respectively). This increase might be attributed to the fact that the use of orthodontic mini-screws as a direct anchorage method made the force action line directed from a wider area (the mini-screw area) to a narrower site (the soldered hooks), which caused the generation of an expanded force that led to the increase in the inter-canine width [35]. In addition, the canines and first molars had moved towards a more expansive area within the dental arch [36].

The present trial results are aligned with the study of Aksakalli et al. [18], while Tuncer et al. [31] found a slight decrease in the inter-canine and inter-molar widths with an average of 0.59 and 0.22 mm, respectively. It might be attributed to the use of a 0.016 × 0.022 in SS archwire. In contrast, in the current study, a 0.019 × 0.025 in SS archwire was used, which provided better control over the movement of the maxillary canines and first molars during the *en-masse* retraction.

There was a slight increase in the periodontal indices following the levelling and alignment phase due to the contribution of fixed orthodontic appliances in the microbial plaque accumulation between the brackets, wires, and other elements [37]. This increase in the periodontal indices was statistically insignificant due to the strict oral hygiene instructions and the close patients' follow-up visits (once every three weeks) [38].

Regarding the periodontal indices after the corticotomy procedures, the differences between the two groups were statistically significant since the periodontal harms were greater in the traditional corticotomy compared to the flapless corticotomy, but clinically the differences were not substantial. No gingival recession was observed in both groups because of the absence of predisposing factors such as severe accumulation of bacterial plaque [39], the application of light forces during levelling and alignment phase, the replacement of wires every three weeks following the accepted principles of orthodontics, and finally, patients' cooperation in maintaining good oral hygiene. No published paper has been found in the literature that has evaluated the periodontal indices in the *en-masse* retraction cases. So, the results obtained in the current work will be compared with studies that have only used the traditional and/or flapless corticotomy to accelerate canine retraction.

The current study agreed with Abbas et al. [22] study in terms of the absence of gingival recession, and it differed from it regarding the gingival, papillary bleeding, and plaque indices as they did not find any significant difference between the control and corticotomy groups due to the difference in the study design. Abbas et al. [22] study was a split-mouth design, and the corticotomy was performed on the buccal side only. The present article agreed with Aboul-Ela et al. [14] regarding the increase in the gingival and plaque indices and differed with them as they found a simple gingival recession. The difference might be attributed to the oral hygiene procedures, as the recession occurred within corticotomy and control groups.

The current study differed from Abed and Al-Bustani [15] as they found no bleeding during probing, and it might be due to elevating the flap only from the buccal side in their study.

Finally, the current article differed with Aksakalli et al. [18] in the gingival index values as they were better within the present study. This indicated the establishment of better oral hygiene in the patients of the current study. Besides, the average age of Aksakalli et al. [18] patients was 16.3 ± 2.4 years, which was associated with hormonal and periodontal changes during adolescence.

All maxillary teeth preserved the vitality of the dental pulp. This finding is consistent with Abbas et al. [22] and Abed and Al-Bustani [15] studies. The reason to maintain the vitality of teeth is performing the incisions only within the cortical bone without interfering with the spongy bone, which is responsible for providing blood supply to the teeth.

### 4.2. Limitations

The focus of the study was directed towards the maxillary arch, and there should be an assessment of the changes in the mandibular arch. The gender effect on the current results was not measured due to the lack of male patients in the study. Besides, there is a need to assess patient-reported outcomes in future research.

### 4.3. Generalizability

The generalizability of the study results may be restricted because the study was carried out on a particular age range, a particular type of malocclusion with rigorous inclusion criteria. In addition, this study was carried out on participants referred to the Orthodontic Department in a teaching hospital and an academic atmosphere. So, more clinical studies must be carried out on different populations with various types of malocclusion to get a better generalization.

## 5. Conclusions

- Both traditional and flapless corticotomy techniques resulted in clinically similar rates of *en-masse* retraction of upper anterior teeth.
- Neither the traditional nor the flapless corticotomy procedure caused a gingival recession at the upper teeth.
- All teeth remained vital following both corticotomies.
- The flapless corticotomy is less invasive than the traditional corticotomy with similar dental and periodontal changes between both techniques.

## Figures and Tables

**Table 1:** Basic characteristics of the sample at the beginning of the treatment.

Group	Traditional corticotomy group (TCG)	Flapless corticotomy group (FCG)
Number of patients	19	19
Gender distribution (male/female)	2/17	1/18
Mean age ± SD (years)	23.84 ± 2.87	23.29 ± 2.94
Crowding (no/minimal)	12/7	11/8
Facial divergence (normal/hyperdivergent)	8/11	10/9
Protrusion of upper anterior teeth (moderate/severe)^*∗*^	4/15	5/14
Overbite (normal/shallow)	7/12	9/10
Posterior crossbite (no/yes)	0/19	0/19

SD: standard deviation. ^*∗*^The protrusion of upper anterior teeth was divided into 3 categories: mild (3–5 mm), moderate (5–7.5 mm), and severe (7.5–10 mm).

**Table 2:** Descriptive statistics of the *en-masse* retraction rate (mm/month) as well as the *P* values of significance tests.

Time	FCG			TCG			FCG vs TCG mean difference (95% CI)	*P* value^†^
	*n*	Mean	SD	*n*	Mean	SD		
Upper central incisors retraction rate								
T0-T1	18	1.60	0.14	18	1.82	0.18	−0.23 (−0.33, −0.12)	<0.001^*∗*^
T1-T2	18	1.42	0.10	18	1.66	0.15	−0.24 (−0.33, −0.16)	<0.001^*∗*^
T2-T3	18	1.22	0.10	18	1.39	0.18	−0.17 (−0.27, −0.07)	0.001^*∗*^
T3-T4	15	1.08	0.08	15	1.14	0.18	−0.06 (−0.16, 0.04)	0.244
T4-T5	9	0.98	0.12	6	0.89	0.11	0.09 (−0.04, 0.22)	0.148
T0-TF	18	6.18	0.25	18	6.84	0.31	−0.65 (−0.84, −0.46)	<0.001^*∗*^

Upper canine retraction rate								
T0-T1	18	1.57	0.19	18	1.80	0.18	−0.23 (−0.35, −0.10)	<0.001^*∗*^
T1-T2	18	1.41	0.10	18	1.63	0.15	−0.22 (−0.31, −0.14)	<0.001^*∗*^
T2-T3	18	1.21	0.10	18	1.38	0.18	−0.17 (−0.27, −0.08)	0.001^*∗*^
T3-T4	15	1.07	0.09	15	1.09	0.22	−0.02 (−0.15, 0.11)	0.742
T4-T5	9	0.98	0.12	6	0.86	0.14	0.11 (−0.03, 0.26)	0.115
T0-TF	18	6.09	0.37	18	6.65	0.40	−0.55 (−0.82, −0.29)	<0.001^*∗*^

^†^Two-sample *t*-test. ^*∗*^Employing Bonferroni's correction for multiplicity, statistical significance was set at *P* < 0.008 (alfa: 0.05/6 = 0.008); FCG: flapless corticotomy group; TCG: traditional corticotomy group; SD: standard deviation; CI: confidence interval; the TCG values were subtracted from the FCG values.

**Table 3:** Descriptive statistics of the upper first molar movement rate (mm/month) as well as the *P* values of significance tests.

Time	FCG			TCG			FCG vs TCG mean difference (95% CI)	*P* value^†^
	*n*	Mean	SD	*n*	Mean	SD		
Right upper first molar movement rate								
T0-T1	18	0.00	0.00	18	0.00	0.03	0.00 (−0.02, 0.01)	0.537
T1-T2	18	0.00	0.00	18	0.00	0.00	0.00 (−0.00, 0.00)	0.946
T2-T3	18	−0.00	0.00	18	0.00	0.00	−0.00 (−0.00, 0.00)	0.067
T3-T4	15	−0.00	0.00	15	−0.08	0.24	0.08 (−0.05, 0.20)	0.207
T4-T5	9	−0.14	0.21	6	−0.19	0.29	0.05 (−0.23, 0.32)	0.733
T0-TF	18	−0.13	0.25	18	−0.18	0.29	0.05 (−0.13, 0.23)	0.589

Left upper first molar movement rate								
T0-T1	18	0.00	0.01	18	0.06	0.27	−0.06 (−0.19, 0.07)	0.348
T1-T2	18	0.00	0.00	18	0.00	0.01	0.00 (−0.01, 0.01)	0.933
T2-T3	18	−0.03	0.11	18	−0.00	0.03	−0.03 (−0.08, 0.03)	0.378
T3-T4	15	−0.02	0.10	15	−0.04	0.14	0.02 (−0.07, 0.11)	0.690
T4-T5	9	−0.16	0.19	6	−0.33	0.32	0.17 (−0.12, 0.45)	0.227
T0-TF	18	−0.16	0.19	18	−0.32	0.32	0.16 (−0.02, 0.34)	0.074

^†^Two-sample *t*-test. Employing Bonferroni's correction for multiplicity, statistical significance was set at *P* < 0.008 (alfa: 0.05/6 = 0.008); FCG: flapless corticotomy group; TCG: traditional corticotomy group; SD: standard deviation; CI: confidence interval; the TCG values were subtracted from the FCG values.

**Table 4:** Descriptive statistics of the inter-canine and inter-molar width changes (mm/month) as well as the *P* values of significance tests.

Time	FCG			TCG			FCG vs TCG mean difference (95% CI)	*P* value^†^
	*n*	Mean	SD	*n*	Mean	SD		
Inter-canine width changes								
T0-T1	18	−0.35	0.16	18	−0.35	0.26	−0.00 (−0.15, 0.14)	0.956
T1-T2	18	−0.34	0.12	18	−0.38	0.13	0.04 (−0.04, 0.12)	0.351
T2-T3	18	-0.31	0.09	18	-0.28	0.22	−0.04 (−0.15, 0.07)	0.482
T3-T4	15	−0.23	0.17	15	−0.31	0.05	0.08 (−0.01, 0.18)	0.091
T4-T5	9	−0.18	0.13	6	−0.28	0.06	0.09 (−0.03, 0.22)	0.140
T0-TF	18	−1.41	0.32	18	−1.59	0.33	0.18 (−0.04, 0.40)	0.108

Inter-molar width changes								
T0-T1	18	0.00	0.00	18	0.00	0.00	−0.00 (−0.00, 0.00)	0.393
T1-T2	18	0.00	0.01	18	0.00	0.01	0.00 (−0.00, 0.01)	0.619
T2-T3	18	−0.04	0.16	18	−0.00	0.03	−0.04 (−0.11, 0.04)	0.324
T3-T4	15	−0.09	0.15	15	−0.08	0.16	−0.02 (−0.13, 0.10)	0.794
T4-T5	9	−0.04	0.19	6	−0.23	0.37	0.19 (−0.12, 0.51)	0.212
T0-TF	18	−0.17	0.24	18	−0.31	0.23	0.15 (−0.02, 0.31)	0.075

^†^Two-sample *t*-test. Employing Bonferroni's correction for multiplicity, statistical significance was set at *P* < 0.008 (alfa: 0.05/6 = 0.008); FCG: flapless corticotomy group; TCG: traditional corticotomy group; SD: standard deviation; CI: confidence interval; the TCG values were subtracted from the FCG values.

**Table 5:** Descriptive statistics of the periodontal indices as well as the *P* values of significance tests.

Time	FCG			TCG			*P* value^†^
	Mean	SD	Median	Mean	SD	Median	
Gingival index							
T0	0.22	0.25	0.12	0.22	0.24	0.16	0.914
T1	0.25	0.22	0.21	0.29	0.13	0.26	0.224
T2	0.28	0.18	0.26	0.44	0.17	0.43	0.003^*∗*^

Papillary bleeding index							
T0	0.14	0.21	0.04	0.14	0.24	0.05	0.935
T1	0.17	0.23	0.08	0.23	0.17	0.21	0.083
T2	0.20	0.19	0.14	0.44	0.28	0.35	0.001^*∗*^

Plaque index							
T0	0.31	0.23	0.32	0.30	0.23	0.28	1.000
T1	0.33	0.28	0.33	0.37	0.18	0.30	0.490
T2	0.38	0.25	0.43	0.59	0.22	0.60	0.002^*∗*^

## Data Availability

The data are available from the corresponding author upon reasonable request.

## Abbreviations

CONSORT: Consolidated Standards of Reporting Trials
FC: Flapless corticotomy
FCG: Flapless corticotomy group
ICCs: Interclass correlation coefficients
NiTi: Nickel-titanium
RAP: Regional acceleration phenomenon
RCT: Randomized controlled trial
SS: Stainless steel
TC: Traditional corticotomy
TCG: Traditional corticotomy group.

## Supplementary Materials

Supplementary Materials Supplementary Table 1: Interclass correlation coefficients of repeated measurements in the current study for the assessment of random error (*n* = 20). Supplementary Table 2: Assessment of the systematic error in the current study (*n* = 20).

## References

[1] Lee J., Miyazawa K., Tabuchi M., Sato T., Kawaguchi M., Goto S. (2014). Effectiveness ofen-masseretraction using midpalatal miniscrews and a modified transpalatal arch: treatment duration and dentoskeletal changes. *Korean journal of orthodontics*.

[2] Langberg B. J., Todd A. (2004). Treatment of a Class I malocclusion with severe bimaxillary protrusion. *American Journal of Orthodontics and Dentofacial Orthopedics*.

[3] Kuroda S., Yamada K., Deguchi T., Kyung H.-M., Takano-Yamamoto T. (2009). Class II malocclusion treated with miniscrew anchorage: comparison with traditional orthodontic mechanics outcomes. *American Journal of Orthodontics and Dentofacial Orthopedics*.

[4] Al-Sibaie S., Hajeer M. Y. (2014). Assessment of changes following en-masse retraction with mini-implants anchorage compared to two-step retraction with conventional anchorage in patients with class II division 1 malocclusion: a randomized controlled trial. *European Journal of Orthodontics*.

[5] Khlef H. N., Hajeer M. Y., Ajaj M. A., Heshmeh O. (2018). Evaluation of treatment outcomes of en masse retraction with temporary skeletal anchorage devices in comparison with two-step retraction with conventional anchorage in patients with dentoalveolar protrusion: a systematic review and meta-analysis. *Contemporary Clinical Dentistry*.

[6] Graber T. M., Vanarsdall R. L., Vig K. W. (1994). *Orthodontics. Current Principles and Techniques*.

[7] Ali F. A., Salman L. H. (2014). Acceleration of canine movement by laser assisted flapless corticotomy an innovative approach in clinical orthodontics. *Journal of Baghdad College of Dentistry*.

[8] Khlef H. N., Hajeer M. Y., Ajaj M. A., Heshmeh O. (2019). En-masse retraction of upper anterior teeth in adult patients with maxillary or bimaxillary dentoalveolar protrusion: a systematic review and meta-analysis. *Journal of Contemporary Dental Practice*.

[9] Alfawal A. M., Hajeer M. Y., Ajaj M. A., Hamadah O., Brad B. (2016). Effectiveness of minimally invasive surgical procedures in the acceleration of tooth movement: a systematic review and meta-analysis. *Progress in Orthodontics*.

[10] Alfawal A. M. H., Hajeer M. Y., Ajaj M. A., Hamadah O., Brad B. (2018). Evaluation of piezocision and laser-assisted flapless corticotomy in the acceleration of canine retraction: a randomized controlled trial. *Head & Face Medicine*.

[11] Khlef H. N., Hajeer M. Y., Ajaj M. A., Heshmeh O., Youssef N., Mahaini L. (2020). The effectiveness of traditional corticotomy vs flapless corticotomy in miniscrew-supported en-masse retraction of maxillary anterior teeth in patients with Class II Division 1 malocclusion: a single-centered, randomized controlled clinical trial. *American Journal of Orthodontics and Dentofacial Orthopedics*.

[12] Alexander S. A. (1991). Effects of orthodontic attachments on the gingival health of permanent second molars. *American Journal of Orthodontics and Dentofacial Orthopedics*.

[13] Ericsson I., Thilander B., Lindhe J. (1978). Periodontal conditions after orthodontic tooth movements in the dog. *Angle Orthodontist*.

[14] Aboul-Ela S. M. B. E.-D., El-Beialy A. R., El-Sayed K. M. F., Selim E. M. N., El-Mangoury N. H., Mostafa Y. A. (2011). Miniscrew implant-supported maxillary canine retraction with and without corticotomy-facilitated orthodontics. *American Journal of Orthodontics and Dentofacial Orthopedics*.

[15] Abed S. S., Al-Bustani A. I. (2013). Corticotomy assisted orthodontic canine retraction. *Journal of Baghdad College of Dentistry*.

[16] Cassetta M., Giansanti M., Di Mambro A., Calasso S., Barbato E. (2016). Minimally invasive corticotomy in orthodontics using a three-dimensional printed CAD/CAM surgical guide. *International Journal of Oral and Maxillofacial Surgery*.

[17] Charavet C., Lecloux G., Bruwier A. (2016). Localized piezoelectric alveolar decortication for orthodontic treatment in adults. *Journal of Dental Research*.

[18] Aksakalli S., Calik B., Kara B., Ezirganli S. (2016). Accelerated tooth movement with piezocision and its periodontal-transversal effects in patients with Class II malocclusion. *Angle Orthodontist*.

[19] Moon C.-H., Wee J.-U., Lee H.-S. (2007). Intrusion of overerupted molars by corticotomy and orthodontic skeletal anchorage. *Angle Orthodontist*.

[20] Wilcko W. M., Wilcko T., Bouquot J. E., Ferguson D. J. (2001). Rapid orthodontics with alveolar reshaping: two case reports of decrowding. *International Journal of Periodontics & Restorative Dentistry*.

[21] Generson R. M., Porter J. M., Zell A., Stratigos G. T. (1978). Combined surgical and orthodontic management of anterior open bite using corticotomy. *Journal of Oral Surgery*.

[22] Abbas N. H., Sabet N. E., Hassan I. T. (2016). Evaluation of corticotomy-facilitated orthodontics and piezocision in rapid canine retraction. *American Journal of Orthodontics and Dentofacial Orthopedics*.

[23] Bass C. C. (1954). The problem of dental health. *Bulletin of the Tulane Medical Faculty*.

[24] Sebaoun J.-D. M., Surmenian J., Dibart S. (2011). Traitements orthodontiques accélérés par piézocision : une alternative mini-invasive aux corticotomies alvéolaires. *L’Orthodontie Française*.

[25] Al Imam G., Ajaj M. A., Hajeer M. Y., Al-Mdalal Y., Almashaal E. (2019). Evaluation of the effectiveness of piezocision-assisted flapless corticotomy in the retraction of four upper incisors: a randomized controlled clinical trial. *Dental and medical problems*.

[26] Silness J., Löe H. (1964). Periodontal disease in pregnancy II. Correlation between oral hygiene and periodontal condition. *Acta Odontologica Scandinavica*.

[27] Löe H., Silness J. (1963). Periodontal disease in pregnancy I. Prevalence and severity. *Acta Odontologica Scandinavica*.

[28] Mühlemann H. R. (1977). Psychological and chemical mediators of gingival health. *Revue D’odonto-stomatologie*.

[29] Miller P. D. (1985). A classification of marginal tissue recession. *International Journal of Periodontics & Restorative Dentistry*.

[30] Sakthi S. V., Vikraman B., Shobana V. R., Iyer S. K., Krishnaswamy N. R. (2014). Corticotomy-assisted retraction: an outcome assessment. *Indian Journal of Dental Research*.

[31] Tunçer N. İ., Arman-Özçırpıcı A., Oduncuoğlu B. F., Göçmen J. S., Kantarcı A. (2017). Efficiency of piezosurgery technique in miniscrew supported en-masse retraction: a single-centre, randomized controlled trial. *European Journal of Orthodontics*.

[32] Vercellotti T. (2004). Technological characteristics and clinical indications of piezoelectric bone surgery. *Minerva Stomatologica*.

[33] Hoggan B. R., Sadowsky C. (2001). The use of palatal rugae for the assessment of anteroposterior tooth movements. *American Journal of Orthodontics and Dentofacial Orthopedics*.

[34] Liu Y. H., Ding W. H., Liu J., Li Q. (2009). Comparison of the differences in cephalometric parameters after active orthodontic treatment applying mini-screw implants or transpalatal arches in adult patients with bialveolar dental protrusion. *Journal of Oral Rehabilitation*.

[35] Cornelis M. A., De Clerck H. J. (2007). Maxillary molar distalization with miniplates assessed on digital models: a prospective clinical trial. *American Journal of Orthodontics and Dentofacial Orthopedics*.

[36] Anuwongnukroh N., Dechkunakorn S., Kunakornporamut K., Tua-Ngam P. (2017). Dental arch changes in postretention in Class II division 1 extraction cases. *International Orthodontics*.

[37] Pandis N., Vlahopoulos K., Madianos P., Eliades T. (2007). Long-term periodontal status of patients with mandibular lingual fixed retention. *European Journal of Orthodontics*.

[38] Davies T. M., Shaw W. C., Worthington H. V., Addy M., Dummer P., Kingdon A. (1991). The effect of orthodontic treatment on plaque and gingivitis. *American Journal of Orthodontics and Dentofacial Orthopedics*.

[39] Melsen B., Allais D. (2005). Factors of importance for the development of dehiscences during labial movement of mandibular incisors: a retrospective study of adult orthodontic patients. *American Journal of Orthodontics and Dentofacial Orthopedics*.

