# Supplementary material for: Is It Possible to Achieve Favorable Accelerated Dental Changes with No Periodontal Complications When Retracting Upper Anterior Teeth Assisted by Flapless Corticotomy Compared to Traditional Corticotomy? A Two-Arm Randomized Controlled Trial
Source: ScientificWorldJournal. 2022 Mar 7;2022:4261248. doi: 10.1155/2022/4261248 (PMC8920673; doi:10.1155/2022/4261248)
Supplement: Supplementary Materials — Supplementary Table 1: Interclass correlation coefficients of repeated measurements in the current study for the assessment of random error (n = 20). Supplementary Table 2: Assessment of the systematic error in the current study (n = 20). [file 4261248.f1.zip › 4261248.f1/Supplementary Table 1.docx]

| Supplementary Table 1: Interclass correlation coefficients of repeated measurements in the current study for the assessment of random error (n=20) | | | | |
| --- | --- | --- | --- | --- |
| Variable | **ICCs †** | **95% Confidence Interval** | | |
|  |  | **minimum** | | **maximum** |
| U1R | 1.000 | 0.999 | 1.000 | |
| U1L | 1.000 | 0.999 | 1.000 | |
| U3R | 0.999 | 0.998 | 1.000 | |
| U3L | 0.999 | 0.998 | 1.000 | |
| U6R | 0.999 | 0.997 | 1.000 | |
| U6L | 0.999 | 0.998 | 1.000 | |
| W13-23 | 0.991 | 0.978 | 0.996 | |
| W16-26 | 0.996 | 0.989 | 0.998 | |
| † Employing ICCs: interclass correlation coefficients. | | | | |
