# Supplementary material for: Is It Possible to Achieve Favorable Accelerated Dental Changes with No Periodontal Complications When Retracting Upper Anterior Teeth Assisted by Flapless Corticotomy Compared to Traditional Corticotomy? A Two-Arm Randomized Controlled Trial
Source: ScientificWorldJournal. 2022 Mar 7;2022:4261248. doi: 10.1155/2022/4261248 (PMC8920673; doi:10.1155/2022/4261248)
Supplement: Supplementary Materials — Supplementary Table 1: Interclass correlation coefficients of repeated measurements in the current study for the assessment of random error (n = 20). Supplementary Table 2: Assessment of the systematic error in the current study (n = 20). [file 4261248.f1.zip › 4261248.f1/Supplementary Table 2.docx]

| Supplementary Table 2:  Assessment of the systematic error in the current study (n=20) | | | | |
| --- | --- | --- | --- | --- |
| Variable | **Mean (SD)**  **1^ST^ measurement** | **Mean (SD)**  **2^nd^ measurement** | **Mean Difference ( SD)** | **P-Value**† |
| U1R | 16.07 (3.19) | 16.08 (3.20) | -0.01 (0.08) | 0.419 |
| U1L | 16.07 (3.19) | 16.08 (3.20) | -0.01 (0.08) | 0.419 |
| U3R | 7.17 (2.44) | 7.19 (2.46) | -0.03 (0.09) | 0.206 |
| U3L | 7.17 (2.44) | 7.19 (2.46) | -0.03 (0.09) | 0.206 |
| U6R | 13.04 (1.80) | 13.02 (1.81) | 0.03 (0.08) | 0.198 |
| U6L | 12.94 (1.81) | 12.96 (1.83) | -0.01 (0.07) | 0.390 |
| W13-23 | 36.28 (1.63) | 36.23 (1.73) | 0.05 (0.22) | 0.312 |
| W16-26 | 43.83 (2.39) | 43.78 (2.38) | 0.05 (0.23) | 0.338 |
| † Employing paired sample *t*-test.  SD: standard deviation. | | | | |
